# Crippled Children's Hampers

**Published:** 1906-09-22

**Authors:** W. P. Treloar

**Affiliations:** Alderman.


### EDITOR'S LETTER-BOX.

[Our Correspondents are reminded that prolixity is a great bar to publication, and that brevity of style and conciseness of statement greatly facilitate early insertion.]

### CRIPPLED CHILDREN'S HAMPERS.

*To the Editor of* The Hospital.

Sir,—As I have every reason to anticipate that my time will be very fully occupied in civic affairs during the ensuing twelve months, and as I am anxious that the continuity of the work which I have now carried on for thirteen years shall be in no way interrupted, I am venturing to ask you to let me make an appeal in your columns a little earlier than usual on behalf of the "Little Cripples' Christmas Hamper Fund."

His Majesty the King has year after year headed the subscription list, and this year, when I stated my special reasons for opening the Fund forthwith, his Majesty graciously sent me his customary cheque. I received with it the appended letter from General Sir Dighton Probyn, Keeper of his Majesty's Privy Purse, dated August 4, 1906.

Buckingham Palace.

Dear Sir William Treloar,—I beg to acknowledge the receipt of your letter of July 31, and thank you for same.

I now write by command of the King to let you know that his Majesty will gladly again this year, as in former years, subscribe to the excellent charity in which you take such kindly interest, and I now enclose a cheque for £10 10s.

I am very glad to see the flourishing condition of the Fund, and to know that this good charity is still to be kept up.

I need scarcely remind you that the main object of the "Little Cripples' Christmas Fund" is to send a hamper of Christmas fare direct to the home of each crippled child in London whose state of health is such that it cannot participate in the Children's Banquet at the Guildhall, which is kindly lent for that purpose by the Corporation.

On the morning of that entertainment the Lord Mayor and sheriffs despatch from the Guildhall Yard a long train of vans which distribute throughout the Metropolis upwards of 7,000 hampers, enabling as many little crippled children to act as hosts of the day, each hamper containing enough good things to feed a family.

This work would not be possible without the aid of the Ragged School Union's Cripple Register, maintained throughout the year by continual verification of addresses, home visitation, and ministration.

The Fund which is raised usually provides a balance to be expended in surgical instruments, artificial limbs, invalid chairs, and other benefits greatly appreciated by the suffering little ones.

May I hope therefore that your readers will enable me to put this Fund on a secure footing once more by early contributions, which may be sent, as in previous years, addressed to me at 69 Ludgate Hill, marked "Little Cripples' Christmas Fund."

Yours faithfully,
W. P. Treloar,
Alderman.

